# Water Extracts of *Moringa oleifera* Leaves Alter Oxidative Stress–Induced Neurotoxicity in Human Neuroblastoma SH-SY5Y Cells

**DOI:** 10.1155/2024/7652217

**Published:** 2024-11-13

**Authors:** Agian Jeffilano Barinda, Wawaimuli Arozal, Harri Hardi, Yulia Ratna Dewi, Muhamad Sadam Safutra, Hee Jae Lee

**Affiliations:** ^1^Department of Pharmacology and Therapeutics, Faculty of Medicine, Universitas Indonesia, Jakarta, Indonesia; ^2^Metabolic, Cardiovascular, and Aging Cluster, Indonesia Medical Education and Research Institute (IMERI), Faculty of Medicine, Universitas Indonesia, Jakarta, Indonesia; ^3^Biomedical Sciences, Faculty of Medicine, Universitas Indonesia, Jakarta, Indonesia; ^4^Department of Pharmacology and Pharmacy, Faculty of Medicine, Pattimura University, Poka Campus, Ambon, Indonesia; ^5^Department of Pharmacology, School of Medicine, Kangwon National University, Chuncheon, Republic of Korea

**Keywords:** antioxidant, apoptotic, *Moringa oleifera*, neuroplasticity, SH-SY5Y cells

## Abstract

*Moringa oleifera* (MO) has been an important plant for food and traditional medicine in Asian countries, including Indonesia. The leaves of these plants are reported to be rich in antioxidants, vitamins, and micronutrients and have been proven to have nootropic properties. Therefore, we investigated whether MO could provide protective effects on SH-SY5Y neuroblastoma cells exposed to H_2_O_2_. In this study, we observed cotreating water-extracted MO leaves on the inhibition of reactive oxygen species (ROS). We found that this treatment enhanced the activities of glutathione peroxidase, catalase, and superoxide dismutase. In addition, it suppressed the mRNA expression levels of apoptotic gene-related genes, specifically Bcl-2 associated protein X (BAX) and caspase 3. Furthermore, it promoted neuroplasticity by increasing the brain-derived neurotropic factor (BDNF) mRNA expression in SH-SY5Y cells. The protein expression of phosphorylated-Akt and phosphorylated-CREB, essential genes in neuroplasticity, was also increased in cells treated with H_2_O_2_ and MO. Therefore, the neuroprotective effects of MO against oxidative stress are attributed to its antioxidant and antiapoptotic properties, as well as its ability to modify the neuronal signaling pathway.

## 1. Introduction

Oxidative stress arises from an inequilibrium between the generation of reactive oxygen species (ROS) and the ability of biological systems to detoxify reactive intermediates [1]. Oxidative stress is associated with the progression of many neurodegenerative disorders, including Alzheimer's disease (AD), Parkinson's disease (PD), and other neurodegenerative diseases. Oxidative stress, which results in the damaging effect of free radicals on neurons, plays a detrimental role in the process of neurodegeneration [2]. ROS toxicity is responsible for causing protein misfolding, activation of glial cells, dysfunction of mitochondria, and ultimately leading to cell apoptosis [3]. Other evidence also showed that oxidative stress overproduction is the main cause of neuronal apoptosis in AD [4, 5]. The brain is vulnerable to oxidative stress due to its high level of activity. The brain also has a high oxygen demand, consuming 20% more oxygen than the remainder of the body. The brain is also rich in redox-active metals (copper and iron) actively involved in ROS generation. Brain cell membranes are also enriched with polyunsaturated fatty acids (PUFAs), making them susceptible to lipid peroxidation [6]. Moreover, neurons are thought to be more susceptible to ROS due to increased oxidative metabolism and a lower number of antioxidative enzymes [7]. Therefore, suppression of ROS generation and inhibition of apoptosis can potentially prevent neurodegeneration [8].

Brain-derived neurotrophic factor (BDNF) is an important neurotrophic factor for protecting neurodegenerative diseases. BDNF may allow AKT (also known as protein kinase B) and CREB (cAMP response element-binding protein) activation to modulate neuroplasticity [9]. Several studies have indicated a correlation between oxidative stress and BDNF in the central nervous system [10]. Antioxidant treatment has the potential to enhance the level of BDNF and neurosynaptic function, thereby promoting cognitive preservation. These data suggest that ROS may affect the BDNF signaling pathway in neurodegenerative diseases [11].

*Moringa oleifera* (MO), a plant belongs to the Moringa family, has been consumed and utilized for both culinary and medicinal purposes in various Asian countries, such as Indonesia, where it is commonly referred to as “kelor.” [12]. The leaves of this plant are reported to be a rich source of antioxidants, vitamins, and micronutrients such as potassium, calcium, phosphorus, iron, vitamins A and D, essential amino acids, beta-carotene, vitamin C, and flavonoids [13]. Several studies have shown that MO has numerous benefits, including analgesic, antipyretic, anti-inflammatory, antiallergic, hepatoprotective, gastroprotectives, anticancer, immunostimulant, and the ability to prevent cardiovascular and metabolic disorder [14]. Notably, the leaf extract also exhibits potential as a cognitive enhancer and neuroprotectant in an animal model of dementia. This effect is most likely achieved by reducing oxidative stress and enhancing cholinergic function [15].

Neuroblastoma cell line SH-SY5Y is frequently used to investigate the molecular and cellular mechanisms involving the effects of PD-associated toxins, perform functional studies of familial PD genes, and evaluate potential protective compounds for PD treatment. This cell line has been a valuable asset for elucidating the molecular complexity of PD [16]. This study is the first report on the investigation of MO water extraction on its antioxidant activity, along with antiapoptotic and neuroplasticity-related genes in the SH-SY5Y cell line. A recent study of MO methanol extraction in 2021 on the SH-SY5Y cell line showed promising results in antioxidant activity. However, it did not analyze antiapoptotic and neuroplasticity-related genes [17].

This study is also the first to examine phosphorylated CREB, a neuroplasticity essential gene, in the leaf extract of Moringa. The result of this study may pave the way for further in vivo investigation of the beneficial effect of MO for neuroprotective agents, especially for neurodegenerative diseases caused by oxidative stress.

## 2. Materials and Methods

### 2.1. MO Extract (MOE) Leaves Materials

The MOE was purchased from PT Javaplant (Indonesia). The extract was produced using aqueous extraction and supplemented with maltodextrin. The details of the herb extraction process are provided in Supporting Figure 1, while Supporting Figure 2 includes the certificate of analysis for the MOE.

### 2.2. Cell Culture Study

SH-SY5Y cells, a human neuroblastoma cell line, were purchased from Elabscience. The cells were maintained in DMEM-F12 medium (Gibco) with 15% fetal bovine serum (FBS) (Gibco) and 1x antibiotic–antimycotic (ABAM, 100x Solution, Gibco) under 37°C and 5% CO2. Cells were plated in the previously coated dishes with the poly-D-lysine (Gibco).

The cells were initially cultured on a 10 cm dish until they reached 80% confluency and then passaged into a 96-well plate for cell viability assay, 12-well plate for mRNA analysis, and 6-well plates for immunoblotting analysis, respectively. Afterward, the SH-SY5Y cells were simultaneously exposed to vehicle, H_2_O_2_, or H_2_O_2_ plus MO for 24 h then further analyzed those cells. Phosphate buffered saline (PBS), pH 7.4 with 0.9% NaCl (9 g/L) (Gibco) and dimethyl sulfoxide (DMSO) < 0.1% (Sigma-Aldrich) were used as the control for MOE and H_2_O_2_ treatment, respectively. In addition, a mixture of PBS and DMSO < 0.1% was used for the vehicle.

### 2.3. Cell Viability Assay

A 96-well plate was used for this assay, and 5000 cells were maintained for each well [18]. MOE or H_2_O_2_ was given at various concentrations from 1 to 100 *μ*g/mL and 0.1 to 50 mM, respectively. In brief, eight wells in each group were treated with a certain dose for 24 h and then the cell viability was analyzed using the Vybrant MTT Cell Proliferation Assay Kit (V-13154) (Thermo Fisher, USA), and the proliferation phenotype was detected by analyzing the absorbance at 570 nm with microplate reader.

### 2.4. Quantitative PCR

SH-SY5Y cells were lyzed and extracted into the RNA sample using a Direct-zolTM RNA Miniprep Plus (Zym Research). The cDNA samples were made from roughly 0.5 *μ*g of total RNA using ReverTra qPCR RT Mastermix/gDNA remover kit (Toyobo). Moreover, cDNA was used with the primers and Thunderbird Sybr qPCR mix (Toyobo) to perform quantitative real time-PCR (qRT-PCR) analysis as previously described [18]. The primers used are shown in Table 1. The samples underwent incubation under the specified conditions: cDNA synthesis was conducted for a duration of 10 min at 50°C, iScript reverse transcriptase inactivation was performed for 5 min at 95°C, and PCR cycling for 40 cycles. During the denaturation phase of PCR cycling, the samples were subjected to a temperature of 95°C for 10 s. In the annealing and extension phase, a temperature of 58°C was maintained for 30 s.

### 2.5. Immunoblotting

Immunoblotting was performed as previously explained with minor adjustment [19]. Protein isolated from SH-SY5Y cells was lyzed in radio-immunoprecipitation assay (RIPA) buffer (Sigma-Aldrich, R0278) containing protease (Sigma-Aldrich, P0044) and phosphatase inhibitors (Sigma-Aldrich, P8340). The protein concentration was equalized with the Bradford method before heating in the sample buffer. Afterward, the samples that contained 40 ng of protein concentration were separated by sodium dodecyl sulfate–polyacrylamide gel electrophoresis (SDS–PAGE) and transferred onto a nitrocellulose membrane. The membranes were blocked with 5% skim milk in TBS-T for 30 min and then probed with the first antibody diluted in blocking buffer overnight at 4°C, followed by an incubation process in secondary antibody diluting in blocking buffer. The targeted signals were visualized with enhanced chemiluminescence (ECL) substrate (BioRad) and detected using Chemiluminescence Alliance 4.7 (Uvitec). The data were then presented in arbitrary units after the signals were quantified by normalizing the phosphorylated with the total protein bands. All antibodies for immunoblotting analysis, such as phosphorylated CREB (CST, #9198), total CREB (CST, #9197), phosphorylated Akt (CST, #9271), total Akt (Cell Signaling; #9272), and Anti Rabbit IgG horseradish peroxidase (HRP) Link-Antibody (CST #7074S), were purchased from Cell Signaling Technology (USA).

### 2.6. Statistical Analysis

All data were presented as the mean ± standard error of the mean (SEM). Two-tailed Student's *t*-test was used to investigate the differences between the two groups. One-way ANOVA followed by Tukey's test was used to identify the differences among more than two groups. A *p* value of less than 0.05 was considered statistically significant. GraphPad Prism 8 (GraphPad Software, Inc.; 2018) was used for all statistical analyses.

## 3. Results

### 3.1. The Effect of MOE on the Viability of H_2_O_2_-Treated SH-SY5Y Cells

**Figure 1:**
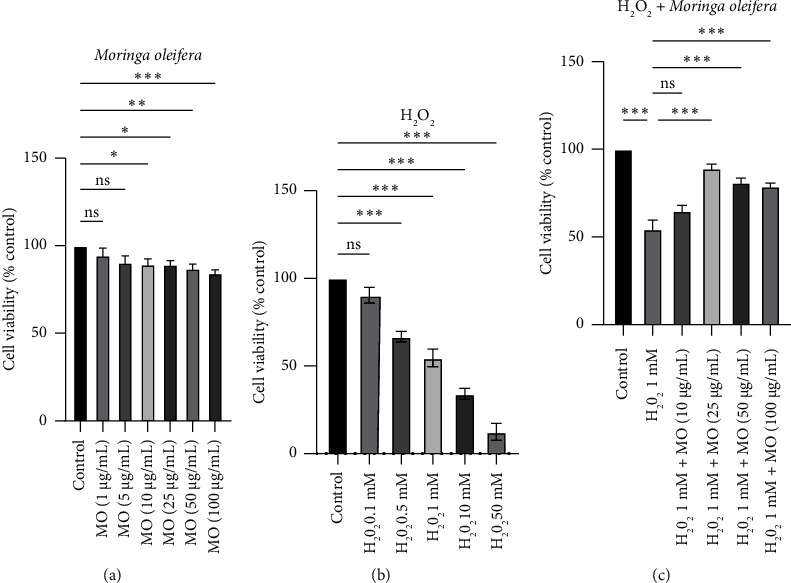
Cell viability assays in SH-SY5Y cells with *Moringa oleifera* (MO) extract and/or H_2_O_2_ treatment. (a) The effect of MO extract alone, ranging from 1 to 100 *μ*g/mL, on cell viability in SH-SY5Y cells. (b) The effect of H_2_O_2_ alone, ranging from 0.1 to 50 mM, on cell viability in SH-SY5Y cells. (c) The effect of H_2_O_2_ concentration at 1 mM and MO extract from 1 to 100 *μ*g/mL on cell viability in SH-SY5Y cells. ns: not significant, ⁣^∗^: *p* < 0.05, ⁣^∗∗^: *p* < 0.01, ⁣^∗∗∗^: *p* < 0.001.

**Figure 2:**
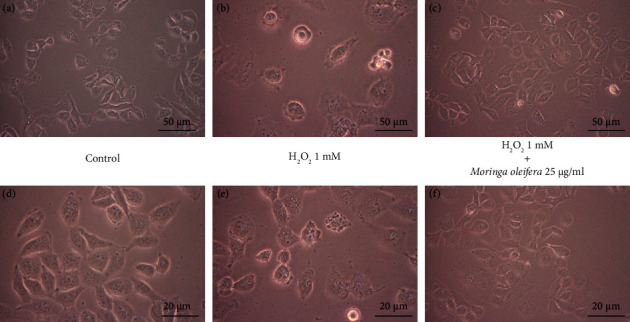
The effects of *Moringa oleifera* (MO) extract with/without H_2_O_2_ treatment on cell morphology in SH-SY5Y cells, with vehicle at 10x magnification (a) and 20x magnification (d), with H_2_O_2_ 1 mM at 10x magnification (b) and 20x magnification (e), and with H_2_O_2_ 1 mM and MO extract 25 *μ*g/mL at 10x magnification (c) and 20x magnification (f).

The initial study was performed to investigate whether MO may induce cytotoxicity at a certain dose. We found that MO treatment did not affect cell viability until MOS at 100 *μ*g/mL (Figure 1(a)). On the other hand, the proliferation phenotype was gradually reduced at H_2_O_2_ treatment in a dose-dependent manner. It was observed that a concentration of 1 mM H_2_O_2_ could inhibit the viability of half of cell population, which is referred to as lethal concentration 50 (LC_50_) (Figure 1(b)). MO can enhance cell viability that has been exposed to H_2_O_2_ in a dose-dependent manner. Among the different concentrations tested, 25 *μ*g/mL of MO showed the highest percentage in cell viability (Figure 1(c)). Therefore, these doses were used for the subsequent studies. Likewise, cell treated with 1 mM H_2_O_2_ showed the shrinkage morphology phenotype (Figures 2(b) and 2(e)) compared with the control group (Figures 2(a) and 2(d)). In addition, cell treated with both MO 25 *μ*g/mL and 1 mM H_2_O_2_ (Figures 2(c) and 2(f)) displayed minimal morphological changes, indicating the protective effect of MO against H_2_O_2_ exposure in SH-SY5Y cells.

### 3.2. The Effect of MOE on Endogenous Antioxidant Enzymes in H_2_O_2_-Treated SH-SY5Y Cells

**Figure 3:**
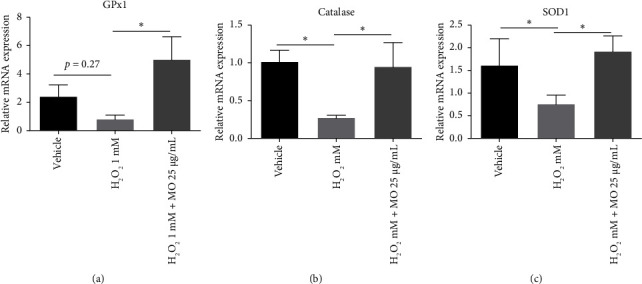
The effects of *Moringa oleifera* (MO) extract on the mRNA expressions of GPx1 (a), catalase (b), and SOD1 (c). Each bar represents the mean relative mRNA expression ± SEM of four samples. Data analysis was performed using one way ANOVA, followed by Tukey's multicomparison test. ns: not significant, ⁣^∗^: *p* < 0.05.

We investigated the antioxidant properties of the MOE, which help maintain the viability phenotype in SH-SY5Y cells following H_2_O_2_ treatment. Several endogenous antioxidant enzymes, such as glutathione peroxidase 1 (GPx1), catalase, and superoxide dismutase (SOD1), were further analyzed [20]. GPx1 tended to be decreased in the H_2_O_2_ group, and MO significantly prevented the inhibition of this mRNA expression level in the H_2_O_2_+MO group (*p* < 0.05) (Figure 3(a)). Similarly, these phenomena were also observed in the catalase and SOD1 mRNA expression (Figures 3(b) and 3(c)).

### 3.3. The Effect of MOE on Apoptotic Markers at the mRNA Level in H_2_O_2_-Treated SH-SY5Y Cells

**Figure 4:**
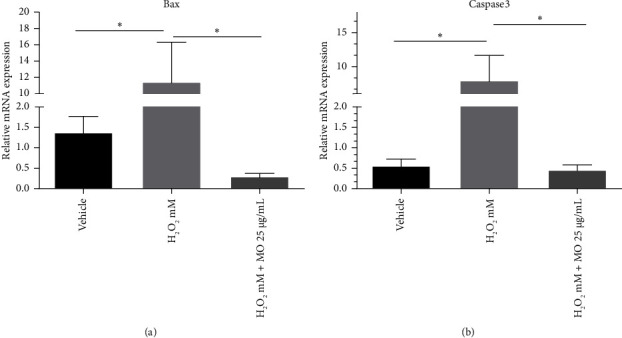
The effects of *Moringa oleifera* (MO) extract on the mRNA expressions of Bax (a) and caspase 3 (b). Each bar represents the mean relative mRNA expression ± SEM of four samples. Data analysis was performed using one way ANOVA, followed by Tukey's multicomparison test. ns: not significant, ⁣^∗^: *p* < 0.05.

We investigated whether MOE extract prevents apoptosis markers in SH-SY5Y cells treated with H_2_O_2_. We found that the H_2_O_2_ group demonstrated the enhancement of Bax and caspase 3 mRNA expression levels compared with the vehicle group (*p* < 0.05). At the same time, MO treatment prevented the increase of these apoptotic markers at the mRNA level (*p* < 0.05) (Figures 4(a) and 4(b)). These data suggest that MOE inhibits Bax and caspase 3 apoptotic activity in SH-S5Y5 cells.

### 3.4. The Effect of MOE on Neuronal Signaling Pathways in H_2_O_2_-Treated SH-SY5Y Cells

**Figure 5:**
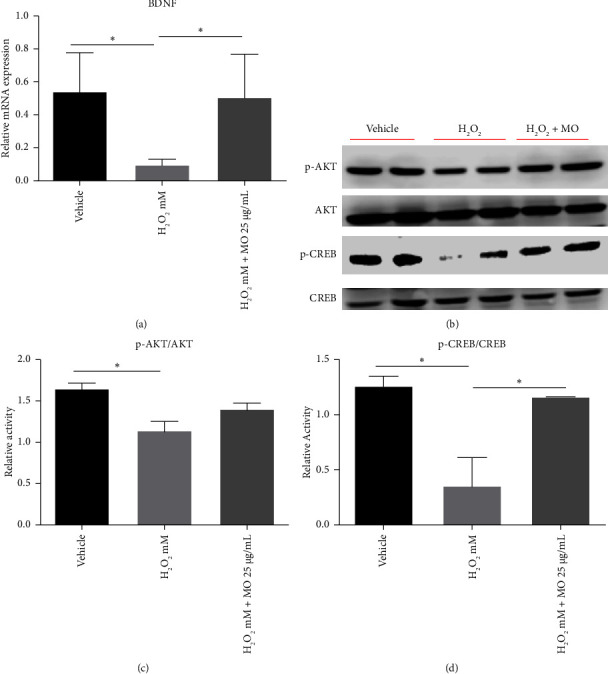
The effects of *Moringa oleifera* (MO) extract on the mRNA expressions of BDNF (a). The representative immunoblotting image in the SH-SY5Y cells of phosphorylated AKT (p-AKT), total AKT (AKT), phosphorylated CREB (p-CREB), and total CREB (CREB) (b). Quantified band analysis of p-AKT protein expression and normalized to AKT protein expression (c). Quantified band analysis of p-CREB protein expression and normalized to CREB protein expression (d). Each bar represents the mean relative mRNA expression ± SEM of four samples. Data analysis was performed using one way ANOVA, followed by Tukey's multicomparison test. ns: not significant, ⁣^∗^: *p* < 0.05.

To elucidate the effect of MOE on the gene involved in neuroplasticity, we analyzed BDNF expression, which plays a vital role in neurogenesis [21]. BDNF showed significantly low expression in the H_2_O_2_ group compared with the vehicle group, and the H_2_O_2_ + MO group was able to enhance the BDNF mRNA expression level (Figure 5(a)). The neurodegenerative diseases have been associated with the dysregulation of AKT and CREB that will impact BDNF gene expression [22]. Cells treated with H_2_O_2_ alone significantly reduced AKT and CREB protein expression. However, MOE treatment appeared to rescue the phosphorylation of the CREB (p-CREB) activity only but not phosphorylation of AKT (p-AKT) activity (Figures 5(b), 5(c), and 5(d)). These data strongly suggest that MO preserves the AKT activity in H_2_O_2_-treated SH-SY5Y cells.

## 4. Discussion

MO is receiving significant attention as a potential treatment for neurodegenerative disease due to its antioxidant properties. Several neuroprotective phytochemicals, such as epigallocatechin gallate, quercetin, gallic acid, and genistein, have been isolated from MO, indicating its neuroprotective properties [23]. The compounds palmitic acid, oleic acid, stearic acid, stigmasterol, and *β* sitosterol exhibited the highest percentages in our previous gas chromatography/mass spectrometry (GC/MS) analysis with MOE from the same manufacturer [24]. This MOE has also been evaluated as a neuroprotective agent in mice with scopolamine-induced memory impairment, demonstrating promising outcomes [24]. Therefore, our study aimed to evaluate the neuroprotective pathway that potentially contributes to the nootropic effect of MO.

Our study found no decreased cell viability from 1 *μ*g/mL to 100 *μ*g/mL MOE concentration. This result is in accordance with another study that found that MOE did not impact cell viability until 100 *μ*g/mL, with cell viability being compromised at 250 *μ*g/mL and 500 *μ*g/mL [17]. Another study showed that moringin, a bioactive compound found in MO, effectively upregulated the expression of p53, p21, and Bax proteins in SH-SY5Y, leading to cell cycle arrest [25]. Moringin also suppressed the activity of nuclear factor kappa-light-chain-enhancer of activated B cells (NF-*κ*B), resulting in a decrease in its viability [25]. These concentration results can be a reference for further in vivo studies to maximize the therapeutic effect and minimize the adverse effect.

In our study, we observed that treating SH-SY5Y cells with a concentration of 25 *μ*g/mL MOE resulted in the greatest improvement in cell viability when exposed to H_2_O_2_-induced cytotoxicity. However, a concentration of 10 *μ*g/mL did not show any significant increase in cell viability. Interestingly, our findings contradict those of a prior investigation, which demonstrated that the viability of cells was enhanced at concentrations of 5, 10, and 25 *μ*g/mL of MOE, with comparable values [17]. The difference in the MO extraction method can cause this contradiction. Our study utilized water extraction, whereas another study utilized methanol extraction. We use water extraction because it contains more alkaloids and saponins than methanol extraction, contributing to better antioxidant properties. However, water extraction has a lower phenol concentration [26, 27]. When choosing between water extraction, ethanol extraction, or other methods of MO extraction, it is important to carefully consider their chemical constituents.

Our study discovered that MOE improved the reduced expression of GPx, caspase, and SOD mRNA following H_2_O_2_ induction. Consistent with our research, a study conducted by Hashim et al. showed that MO leaves exhibited the most potent antioxidant activity compared to other plants examined. This effect was likely attributed to the high amount of polyphenolic compounds in MO leaves [28]. Extensive research has shown that a significant number of polyphenolic compounds can exert beneficial effects on neuroprotection by reducing inflammation, oxidative stress, and protein fibrillation [29]. Therefore, our study confirmed that MOE has capabilities to increase endogenous antioxidant enzymes.

MOE treatment significantly decreased Bax and caspase 3 mRNA expressions in H_2_O_2_-induced SH-SY5Y cells compared with the control group. In line with our study, concurrent treatment with MOE ameliorated oxidative stress, inflammation, and apoptosis in the brain cortex of rats exposed to lead acetate [30]. While an appropriate dose of MO reduces caspase expression, high dose would increase the expression of both caspases 3 and 9, indicating that MO could be toxic in high dose [25]. Caspase could induce chromatic condensation, DNA fragmentation, and blebbing, triggering an intrinsic apoptotic cascade [31]. Hence, MOE has the capacity to decrease the apoptotic markers in a neurotoxicity model with appropriate dosage, suggesting its role as a neuroprotective agent in neurons.

MOE cotreatment with H_2_O_2_ was observed to enhance the BDNF mRNA expression compared with cells treated with H_2_O_2_ alone. The activation of Akt and CREB may account for the increased BDNF expression, as shown by the increasing band density of the MOE group on the western blot. BDNF will activate signaling brain plasticity pathways such as phosphoinositide-3-kinase–protein kinase B/protein kinase B (PI3K/Akt), phospholipase C/inositol trisphosphate/Ca^2+^/calmodulin-dependent protein kinase II (PLC/IP3/CAMKII), and mitogen-activated protein kinase/extracellular signal-regulated kinase 1/2 (MAPK/Erk) pathways, which will increase synaptogenesis and neurogenesis [32, 33]. Our findings are consistent with those of a previous study indicating that MO can reverse the decline of CREB, albeit using a different MO preparation and cell model [34]. The effect of MO on the AKT pathway has been investigated in numerous studies employing various models with promising results [35–37].

Some limitations are needed to be addressed in this study. This study aims to investigate the efficacy of MOE in protecting SH-SY5Y cells after oxidative stress exposure. The herb company made the MOE and provided it to us. Therefore, we did not analyze the characterization or the chemical compositions of the extract. Further studies are necessary to be performed by using GC/MS or liquid chromatography mass spectrometry/mass spectrometry (LC-MS/MS) to analyze those issues. Moreover, future studies are also needed to perform endogenous antioxidant enzymes such as GPx, catalase, or SOD to further investigate those enzyme activities more in details. Another limitation of this study is the lack of experimental replicates, which could impact the reproducibility of the results. Future studies should address this by including multiple replicates to confirm the findings.

In conclusion, we revealed that the water extract of MO has neuroplasticity potential by upregulating BDNF expression via activating the Akt and CREB signaling pathways. We postulated that these effects were due to MOE's antioxidant (increasing GPx1, catalase, and SOD1 expressions) and antiapoptotic (decreasing Bax and caspase 3 expressions) properties.

## Figures and Tables

**Table 1:** Primers for quantitative real-time PCR.

Gene	Primer	Sequence
*β*-actin	Forward	TTGCGCTCAGGAGGAGCAAT
	Reverse	TTCCAGCCTTCCTTCCTGG

Caspase 3	Forward	GGTTAACCCGGGTAAGAATGTGCA
	Reverse	TCGGTCTGGTACAGATGTCGAT

Bax	Forward	CATGTTTTCTGACGGCAACTTC
	Reverse	AGGGCCTTGAGCACCAGTTT

SOD1	Forward	CCACACCTTCACTGGTCCAT
	Reverse	CTAGCGAGTTATGGCGACG

GPx1	Forward	TCGGCTTCCCGTGCAACCAG
	Reverse	CGCACCGTTCACCTCGCACTT

*Catalase*	Forward	TGGTAAACTGGTCTTAAACCGGAATC
	Reverse	GGCGGTGAGTGTCAGGATAGG

BDNF	Forward	CATCCGAGGACAAGGTGGCTTG
	Reverse	GCCGAACTTTCTGGTCCTCATC

## Data Availability

The data that support the findings of this study are available from the corresponding author upon reasonable request.

## Supporting Information

Supporting Figure 1: Flowchart illustrating the water extracts of *Moringa oleifera* leaves process. (a) Extraction to evaporation process. (b) Drying to packing process. Supporting Figure 2: Water extracts of *Moringa oleifera* composition.
