# Supplementary material for: Water Extracts of Moringa oleifera Leaves Alter Oxidative Stress–Induced Neurotoxicity in Human Neuroblastoma SH-SY5Y Cells
Source: ScientificWorldJournal. 2024 Nov 13;2024:7652217. doi: 10.1155/2024/7652217 (PMC11578659; doi:10.1155/2024/7652217)

**Supplementary Figure 1:** Flowchart illustrating the Water extracts of *Moringa oleifera* leaves process. (a) Extraction to evaporation process. (b) Drying to packing process.


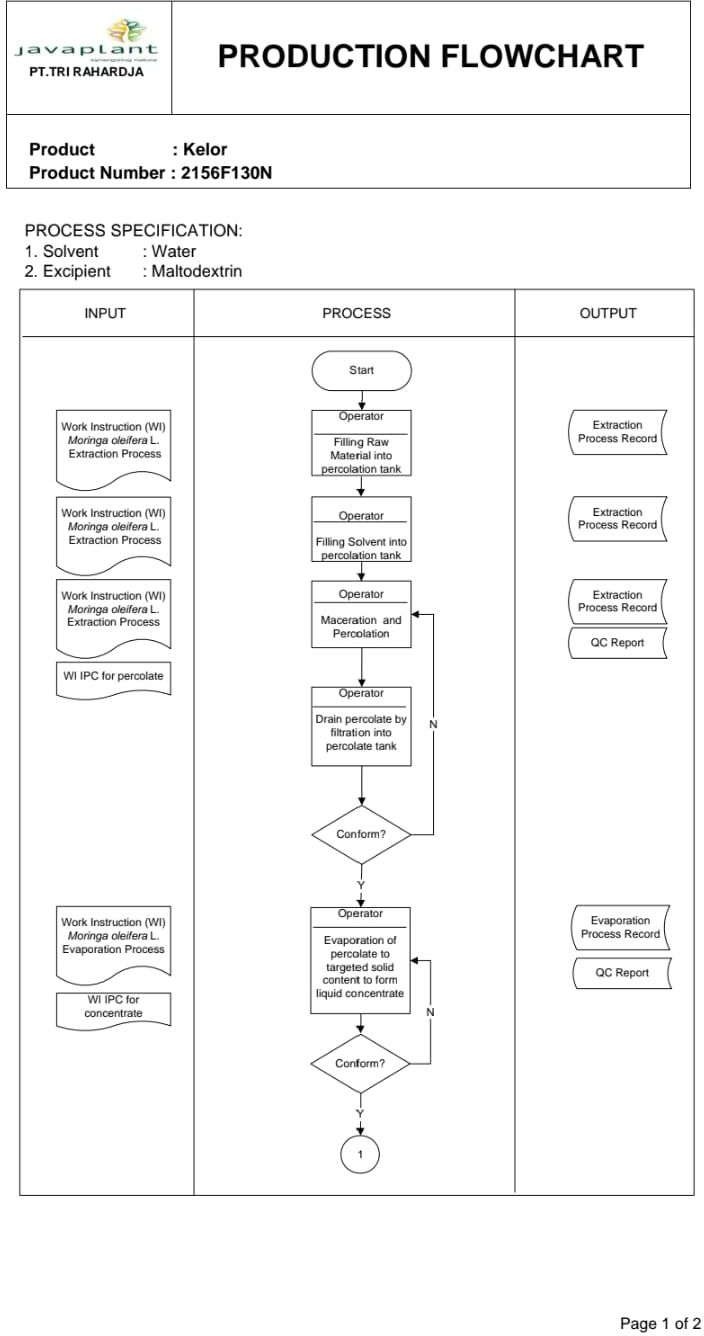


(a)


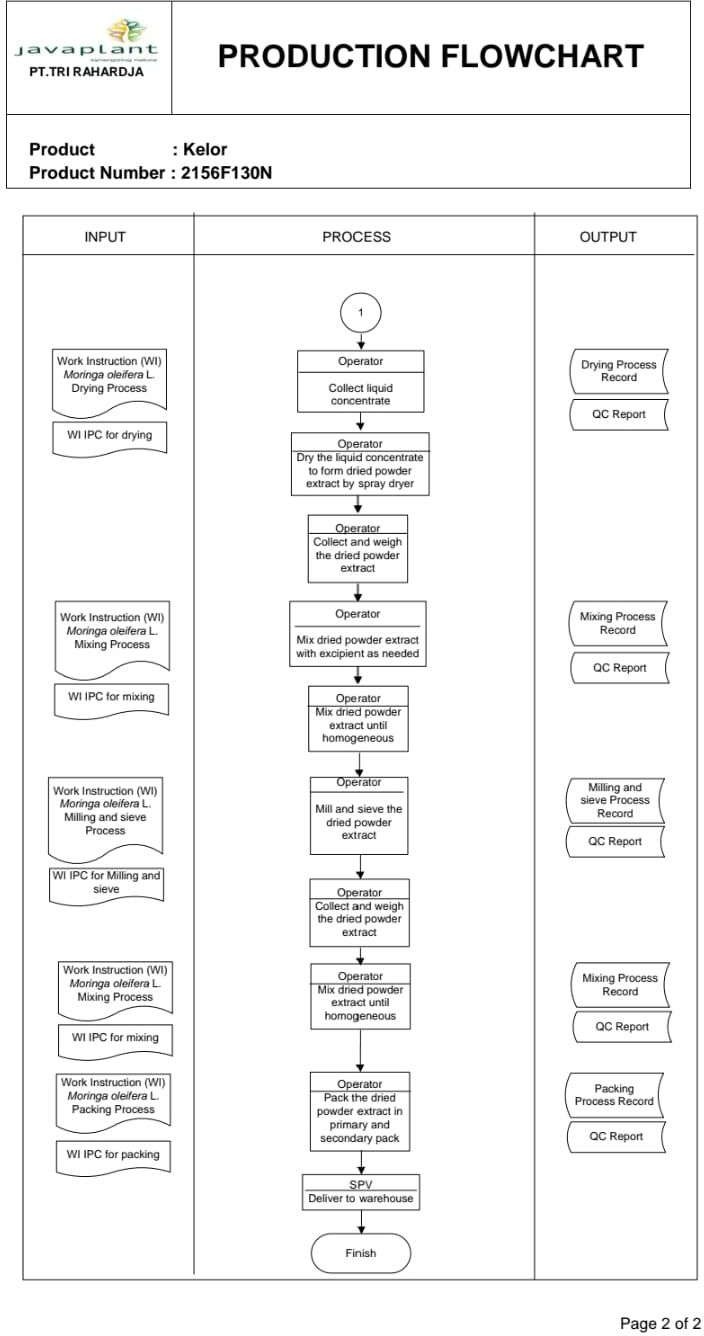
(b)

**Supplementary Figure 2:**  Water extracts of *Moringa oleifera* composition


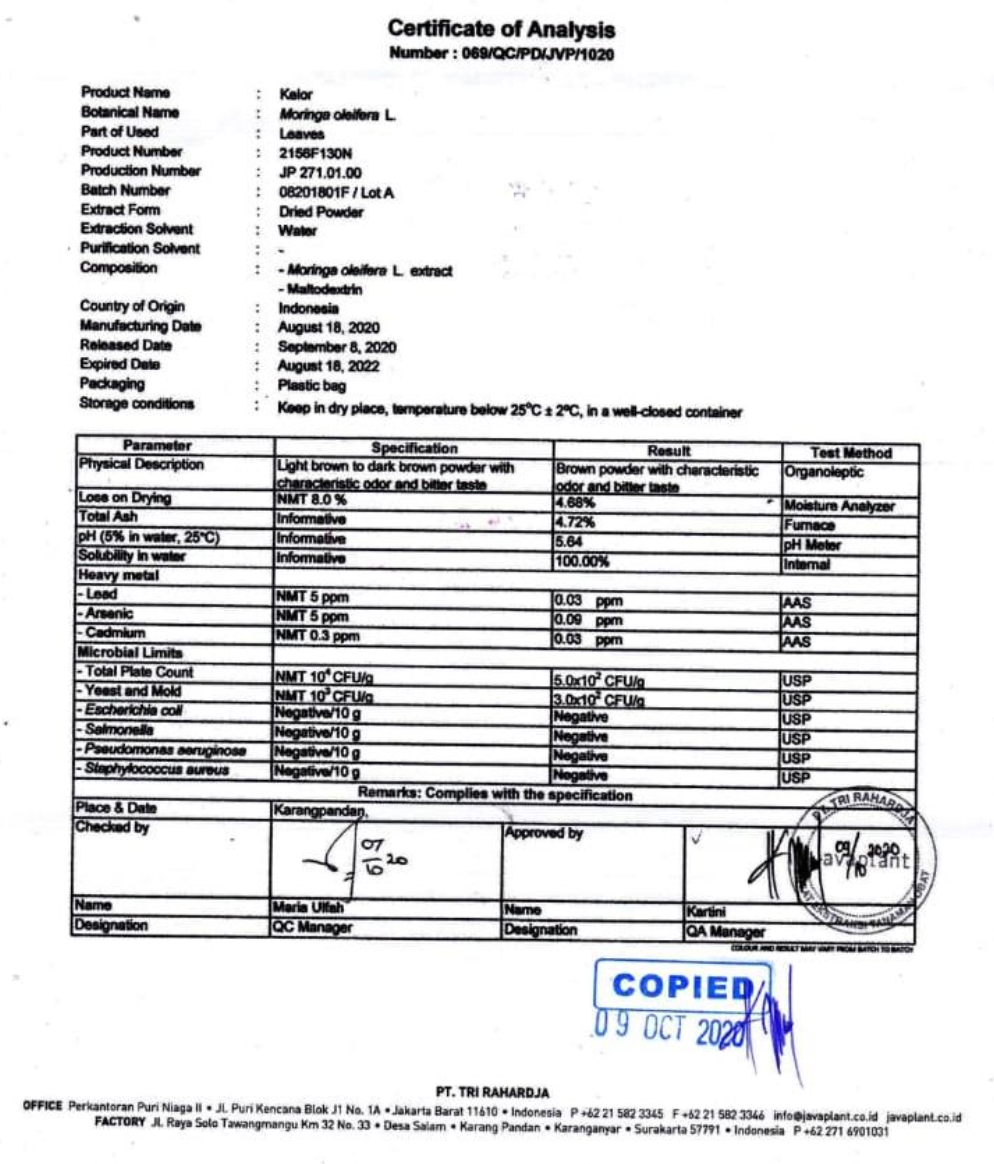

Supplement: Supporting Information — Additional supporting information can be found online in the Supporting Information section. [file 7652217.f1.docx]
